# Distinguishing stroke from transient ischemic attack using plaque characteristics and arterial transit artifacts

**DOI:** 10.3389/fneur.2025.1514679

**Published:** 2025-03-21

**Authors:** Ling Li, Peichun Pan, Na Zhang, Yu Wen, Min Tang, Kai Ai, Xiaoling Zhang, Xiaoyan Lei, Xuejiao Yan

**Affiliations:** ^1^Shaanxi Provincial People's Hospital, Xi'an, China; ^2^Faculty of Medical Technology, Shaanxi University of Chinese Medicine, Xianyang, China; ^3^Department of Clinical Science, Philips Healthcare, Xi’an, China

**Keywords:** arterial spin labeling, arterial transit artifacts, high-resolution MRI, transient ischemic attack, stroke

## Abstract

**Purpose:**

We aimed to investigate the differences in plaque characteristics and hemodynamics in patients with ischemic stroke and transient ischemic attack (TIA), comparing the diagnostic abilities of high-resolution magnetic resonance imaging (HRMRI) and arterial spin labeling (ASL) for ischemic stroke.

**Methods:**

This retrospective analysis included patients who underwent HRMRI and ASL between October 2020 and December 2023. We compared clinical risk factors, vascular plaque characteristics, and the presence of arterial transit artifacts (ATAs) at post-labeling delays (PLDs) of 1.5-s and 2.5-s between stroke and TIA groups. Multivariate logistic regression analysis was used to evaluate the diagnostic performance of different prediction models combining clinical factors, differential plaque characteristics, and the presence of ^PLD ATAs.

**Results:**

A total of 147 patients (mean age, 57.12 ± 13.08 years; 102 men) were initially included in this study, divided into stroke (79) and TIA (68) groups. Significant differences in vascular positive remodeling, intraplaque hemorrhage, enhancement ratio, and the presence of 1.5-s and 2.5-s ATAs (*p* < 0.05) were observed between groups. Combined HRMRI and ASL performed best in distinguishing ischemic stroke and TIA (area under the curve [AUC], 0.926; 95% confidence interval [CI], 0.885–0.967), with no significant difference in ischemic stroke diagnostic performance between HRMRI and ASL (95% CI, −0.039 to 0.087, *Z* = 0.742, *p* = 0.458).

**Conclusion:**

A model combined with plaque characteristics and ATAs showed good diagnostic performance in distinguishing between TIA and stroke in patients with intracranial atherosclerotic stenosis. ASL provides a simpler imaging evaluation method than HRMRI, and ATA evaluation may become a more widely used imaging marker in clinical practice.

## Introduction

Ischemic stroke is a common cerebrovascular disease which has become one of the main causes of disease-related death in China (1, 2). Intracranial atherosclerotic stenosis (ICAS), which most commonly involves the middle cerebral artery (MCA), is the main cause of transient ischemic attack (TIA) and ischemic stroke (3, 4). TIA is a clinical precursor to the onset of ischemic stroke: approximately 7.5 to 17.4% of patients with TIA develop an ischemic stroke within 3 months (5, 6). Therefore, differences in plaque characteristics and hemodynamics between patients with ischemic stroke and TIA must be evaluated. This facilitates clinical risk stratification to guide specific treatment in patients with symptomatic atherosclerotic stenosis.

In clinical practice, the standard diagnostic approach for intracranial atherosclerotic stenosis (ICAS) primarily relies on vascular imaging to evaluate the degree of stenosis and plaque morphology (4). High-resolution magnetic resonance imaging (HRMRI) can non-invasively characterize lumen and plaque features of intracranial atherosclerosis, such as plaque morphology and plaque burden, which are associated with ischemic events (4, 7, 8). However, patients with ICAS rarely undergo MRI assessments using techniques for evaluating hemodynamic injuries, such as dynamic sensitivity contrast-enhanced (DSC) perfusion-weighted imaging (PWI) or arterial spin labeling (ASL) (9, 10). Consequently, studies distinguishing the plaque and hemodynamic features in patients with TIA and irreversible infarction are limited. Although DSC-PWI findings are associated with neurological impairment (11), DSC-PWI is an invasive procedure that requires complex post-processing, making it cumbersome and unsuitable for patient populations such as those with renal dysfunction (12).

Unlike DSC-PWI, ASL allows convenient and non-invasive visual assessment of arterial transport artifacts (ATAs), which occur when labeled blood remains in the supplying arteries/arterioles without being distributed to the microvascular system and tissues. These artifacts appear as bright signals in blood vessels on the surface of the brain and can indicate collateral blood flow and arterial stenosis/occlusion (13). Two single ASL scans performed with different post-labeling delays (PLDs), such as 1,500 and 2,500 ms, can visualize collateral circulation, providing valuable information and facilitating diagnoses (14, 15).

A multimodal approach combining plaque characteristics, hemodynamic parameters, and clinical risk factors may help to understand the mechanism of stroke occurrence. However, studies of differences between TIA and stroke patients have focused on plaque characteristics, and few studies have used ASL for assessment (11). We aimed to investigate differential characteristics of plaque in MCA and hemodynamics in patients with ischemic stroke and TIA and to compare the ability of HRMRI and ASL with two PLDs to predict the occurrence of ischemic stroke.

## Materials and methods

### Ethics

The study was approved by the ethics committee of our institution and was performed in accordance with the tenets of the 1964 Declaration of Helsinki and its later amendments or comparable ethical standards. The need for informed consent was waived.

### Patients

The data of symptomatic ICAS patients with moderate-to-severe MCA stenosis who underwent intracranial HRMRI and ASL examinations between October 2020 and December 2023 were selected from the hospital database for retrospective analysis.

**Figure 1:**
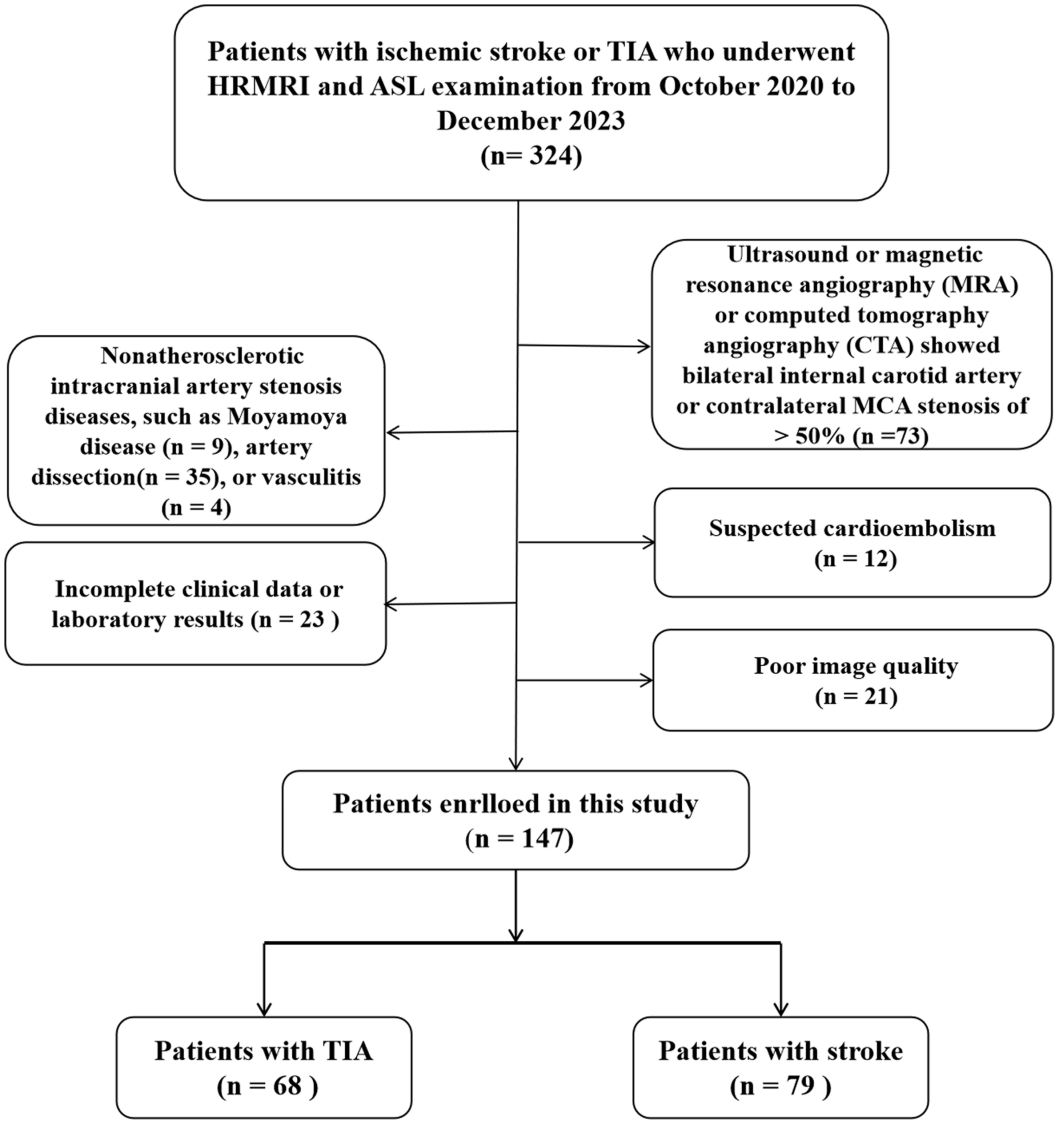
Flowchart of the study population. TIA, transient ischemic attack; HRMRI, high-resolution magnetic resonance imaging; ASL, arterial spin labeling.

The inclusion criteria were as follows: (1) clinical and imaging diagnosis of acute or subacute ischemic stroke or TIA in the MCA region within 2 weeks of symptom onset; (2) unilateral MCA stenosis detected in time-of-flight magnetic resonance angiography (TOF-MRA); and (3) at least one intracranial atherosclerotic plaque. Exclusion criteria were as follows: (1) ultrasound or magnetic resonance angiography or computed tomography angiography revealed a bilateral internal carotid artery or contralateral MCA stenosis of >50%; (2) non-ICAS diseases such as Moyamoya disease, arterial dissection, or vasculitis; (3) suspected cardioembolism; (4) incomplete clinical data or laboratory results; and (5) unsatisfactory image quality. A previously established 4-point scale was used to assess the MRI quality (1 = poor quality, 2 = acceptable, 3 = good quality, 4 = excellent). Images rated as poor quality were excluded from the final study. Figure 1 provides a flowchart of inclusion and exclusion criteria.

The patients were classified into stroke and TIA groups according to the results of diffusion-weighted imaging (DWI) and neurological examinations. TIA was defined as a transient episode of neurological dysfunction caused by focal cerebral ischemia that is not accompanied by acute cerebral infarction. In contrast, acute ischemic stroke was defined as sudden neurologic dysfunction caused by focal brain ischemia lasting more than 24 h or with evidence of acute infarction on brain imaging and diffusion restriction on DWI (6). Among the 147 patients included in the study, 79 and 68 were categorized into the ischemic stroke and TIA groups, respectively.

### Clinical data collection

The following clinical information was obtained: age; sex; smoker; BMI; hypertension [self-reported high blood pressure history, mean blood pressure > 140/90 mmHg, or the use of antihypertensive medication (16)]; diabetes [patients using insulin or oral hypoglycemic agents or newly diagnosed with diabetes based on hemoglobin A1C ≥6.5% (17)]; hyperlipidemia [total cholesterol ≥5.2 mmol/L, triglycerides ≥1.7 mmol/L, high-density lipoprotein cholesterol ≤1.0 mmol/L, low-density lipoprotein cholesterol ≥3.4 mmol/L (18)]; hyperhomocysteinemia (homocysteine ≥15 μmol/L); and history of coronary artery disease (19).

### MRI protocol

Imaging was performed on a 3.0-T Philips Ingenia CX scanner. The scan protocols included a head MRI routine examination (T1-weighted imaging [T1WI], T2-weighted imaging [T2WI], and fluid attenuation inversion recovery) and DWI examination. HRMRI was performed within 1 week of symptom onset, including three-dimensional (3D)-TOF MRA, 3D-T1WI-Volume-isotropic TSE acquisition (VISTA), T2WI-Turbo Spin Echo (TSE), 3D-T1-Vista enhanced imaging, and 2-PLD ASL-PWI. The parameters of these sequences are provided in Table 1.

**Table 1:** Imaging parameters for each sequence.

Imaging parameters	TR (ms)	TE (ms)	FOV (mm)	Matrix (mm)	Slice thickness (mm)	Slice gap (mm)
T1WI	2000	20	230 × 180	512 × 512	6	1
T2WI	2,500	80	230 × 180	512 × 512	6	1
FLAIR	6,000	120	230 × 180	480 × 480	6	1
DWI	2,562	94	230 × 230	224 × 224	6	1
TOF-MRA	20	3.6	180 × 180	512 × 512	0.5	0
3D-T1WI-VISTA	700	16	180 × 180	256 × 256	0.6	0
T2WI-TSE	3,000	90	140 × 100	288 × 288	2.0	0.2
1.5 s-ASL	3,935	9.8	240 × 240	80 × 80	6.0	0
2.5 s-ASL	3,935	9.8	240 × 240	80 × 80	6.0	0

TR, repetition time; TE, echo time; FOV, field of view. Post-contrast T1WI was performed 5 min after injecting a single dose (0.1 mmol/kg of body weight) of gadolinium-based contrast agent (Magnevist; Bayer HealthCare Pharmaceuticals).

### Image analysis

All MRI data were transferred to semiautomatic software^1^ for analysis. Plaque characterization and ASL visual assessment were performed on a workstation by two experienced neuroradiologists (with 10 and 6 years of experience in neuroimaging diagnosis, respectively) who had no knowledge of the patient’s clinical information or previous imaging data. For qualitative assessments, a third senior neuroradiologist (with 17 years of experience) was consulted to review the images and help reach a consensus. For quantitative evaluations, the average of the measurements from the two radiologists was used in the final analysis. Atherosclerotic plaque was defined as significantly eccentric or focal thickening of the vessel wall on reconstructed HRMRI images, with the thinnest wall ≤50% of the thickest (20). The plaque characteristics of MCA maximum lumen stenosis (MLN) were analyzed. First, the post-contrast T1WI images were reconstructed using a multi-plane reconstruction tool, according to the vascular orientation of the MLN site. Then, the inner and outer walls of the lumen on the short-axis reconstructed image were manually sketched using post-processing software. The reference site was the lumen without plaque closest to the proximal or distal end of the MLN site. Maximum wall thickness, total vascular area, and lumen area were measured three times and averaged for final statistical analysis. The plaque characteristics were evaluated as follows (21, 22):

a.  Plaque burden = [1 − lumen area_(MLN)_/total vessel area_(MLN)_] × 100%.
b.  Degree of stenosis = [1 − lumen area_(MLN)_/lumen area_(reference)_] × 100%.
c.  Remodeling index = total vessel area_(MLN)_/total vessel area_(reference)_.

Remodeling index ≥1.05 and < 1.05 indicated positive and non-positive remodeling, respectively.

d.  Eccentricity index = maximum wall thickness – minimum wall thickness/maximum wall thickness.
e.  Intraplaque hemorrhage was defined as the T1WI signal in the plaque being ≥150% of the T1WI signal in the adjacent muscle or pons.
f.  Enhancement was assessed by comparing pre- and post-enhancement HRMRI. Plaque enhancement was classified into three grades: grade 2 (enhancement equal to or greater than the pituitary funnel), grade 1 (enhancement greater than the normal intracranial vascular wall but less than the pituitary funnel), and grade 0 (enhancement equal to or less than the normal intracranial vascular wall). The enhanced ratio was estimated as follows: enhanced ratio = (Signal_post_ − Signal_pre_)/Signal_pre_.
g.  ATAs were defined by the presence of highlighted signals in blood vessels covering the surface of the brain. Our study used ASL of two PLDs (1.5 s and 2.5 s) and evaluated the presence of ATAs on two PLD (hereafter referred to as 2-PLD ATAs) images (10).

### Statistical analysis

The normality of continuous variables was assessed using the Shapiro–Wilk test. Normally distributed data are presented as mean ± standard deviation and analyzed with the Student’s *t*-test, while non-normally distributed data are presented as median (25th–75th percentile) and analyzed using the Mann–Whitney U-test. Categorical variables are presented as counts and percentages and compared using the chi-square test or Fisher’s exact test when the expected frequency was below 5. Inter-reader reliability was analyzed using intraclass correlation efficient (ICC) values. Variables were selected for multivariate analysis through (1) statistical significance threshold (*p* < 0.05) in univariate analyses, and (2) variables with established biological or clinical relevance, such as age and gender, were included in the multivariate analysis, regardless of their univariate statistical significance. Models using different combinations of risk factors were constructed, and the diagnostic performance of each model was assessed using receiver operating characteristic (ROC) curves. The Z-test was used to compare the area under the curve (AUC) of the different models. The above statistics were two-sided and performed using SPSS (version 26, IBM) or R (version 4.0.4, R Foundation for Statistical). A *p* < 0.05 is considered statistically significant.

## Results

### Patient demographics and clinical characteristics

A total of 147 patients (mean age, 57.12 ± 13.08 years; 29–88 years, 102 men) were initially included in this study, including 68 patients (59.82 ± 13.38 years, 46 men) in the TIA group and 79 patients (54.79 ± 12.44 years, 56 men) in the stroke group. Demographic and clinical characteristics of the enrolled patients are presented in Table 2. Compared with TIA patients, stroke patients were younger and had a significantly higher prevalence of hypertension (*p* = 0.001). The remaining clinical characteristics showed no significant differences between the two groups.

**Table 2:** Demographic and clinical characteristics of the enrolled patients.

Characteristics	Total sample (*n* = 147)	TIA group (*n* = 68)	Stroke group (*n* = 79)	*p-*value
Age (years)	57.12 ± 13.08	59.82 ± 13.38	54.79 ± 12.44	0.021^a^^,^*
Male sex	102 (69.39)	46 (67.65)	56 (70.89)	0.806
Smoker	92 (62.59)	46 (67.65)	46 (58.23)	0.315
BMI	24.38 [22.51, 26.08]	24.80 [22.62, 26.44]	24.00 [22.51, 25.89]	0.367
Hypertension	95 (64.63)	35 (51.47)	60 (75.95)	0.003*
Diabetes mellitus	50 (34.01)	24 (35.29)	26 (32.91)	0.926
HbA1c	5.90 [5.60, 6.55]	5.90 [5.60, 6.73]	5.80 [5.50, 6.40]	0.227
Hyperlipidemia	62 (42.18)	24 (35.29)	38 (48.10)	0.161
TC (mmol/L)	3.70 [3.15, 4.63]	3.76 [3.12, 4.70]	3.70 [3.20, 4.26]	0.556
TG (mmol/L)	1.21 [0.95, 1.60]	1.21 [0.94, 1.78]	1.17 [0.96, 1.43]	0.281
LDL (mmol/L)	0.99 [0.86, 1.20]	1.03 [0.90, 1.20]	0.93 [0.84, 1.10]	0.075
HDL (mmol/L)	2.21 [1.71, 2.73]	2.20 [1.70, 2.82]	2.23 [1.77, 2.66]	0.804
Hyperhomocysteinemia	73 (49.66)	29 (42.65)	44 (55.70)	0.158
Homocysteine (μmol/L)	15.60 [12.80, 24.00]	15.25 [12.80, 24.00]	15.80 [12.95, 23.85]	0.659
History of coronary artery disease	29 (19.73)	9 (13.24)	20 (25.32)	0.104

All values are presented as number (%) or median [interquartile range] unless otherwise indicated. HbA1c, glycosylated hemoglobin; TC, total cholesterol; TG, triglyceride; HDL, high-density lipoprotein cholesterol; LDL, low-density lipoprotein cholesterol. * *p* < 0.05 indicates a significant difference between the two groups.

a Data presented as mean ± standard deviation.

### Inter-reader agreement

Measurement of plaque characteristics and ASL showed good-to-excellent inter-reader agreement (ICC = 0.796–0.966; *p* < 0.001), as shown in Supplementary Table 1.

### Intergroup differences in plaque characteristics and the presence of 2-PLD ATAs

The comparison of plaque characteristics and the presence of 2-PLD ATAs between the two groups are shown in Table 3. In the stroke group, plaque burden (*p* < 0.001), degree of stenosis (*p* < 0.001), remodeling index (*p* < 0.001), the prevalence of positive remodeling (*p* < 0.001) and intraplaque hemorrhage (*p* < 0.001), enhanced ratio (*p* < 0.001), and enhanced grade (*p* < 0.001) were significantly higher than in the TIA group. The presence of 1.5-s ATAs and 2.5-s ATAs was more common in the stroke group than in the TIA group (*p* < 0.001).

**Table 3:** Comparison of HRMRI and ASL characteristics in the two groups.

Characteristics	Total sample (*n* = 147)	TIA group (*n* = 68)	Stroke group (*n* = 79)	*p-*value
Plaque burden (%)	41.72 ± 14.92	33.77 ± 14.79	48.57 ± 11.23	<0.001*^,a^
Degree of stenosis (%)	72.62 [56.09, 82.45]	62.06 [48.76, 77.77]	77.64 [68.68, 86.63]	<0.001*
Remodeling index	1.06 [0.98, 1.19]	1.03 [0.92, 1.08]	1.13 [1.02, 1.23]	<0.001*
Positive remodeling	76 (51.70)	20 (29.41)	56 (70.89)	<0.001*
Eccentricity index	0.59 ± 0.15	0.60 ± 0.14	0.58 ± 0.16	0.628^a^
Intraplaque hemorrhage	44 (29.93)	8 (11.76)	36 (45.57)	<0.001*
Enhancement ratio (%)	42.77 [17.58, 73.37]	20.52 [7.25, 42.92]	59.88 [35.28, 90.35]	<0.001*
Enhanced grade (%)				<0.001*
Grade 0	21 (14.29)	18 (26.47)	3 (3.80)	
Grade 1	58 (39.46)	42 (61.76)	16 (20.25)	
Grade 2	68 (46.26)	8 (11.76)	60 (75.95)	
1.5-s ATAs present (%)	105 (71.43)	33 (48.53)	72 (91.14)	<0.001*
2.5-s ATAs present (%)	69 (46.94)	11 (16.18)	58 (73.42)	<0.001*

All values are presented as numbers (%) or median [interquartile range] unless otherwise indicated. HRMRI, high-resolution magnetic resonance imaging; TIA, transient ischemic attack; ATA, arterial transit artifacts. * *p* < 0.05 indicates a significant difference between the two groups.

a Data presented as mean ± standard deviation.

### The ability of plaque characteristics and ASL visual assessment to differentiate ischemic stroke

In the univariate analysis, plaque burden, degree of stenosis, positive remodeling, intraplaque hemorrhage, enhancement ratio, and the presence of 1.5-s and 2.5-s ATAs were associated with stroke (*p* < 0.05, Table 4). We combined the differential plaque characteristics and the presence of 2-PLD ATAs with clinical factors to establish three models: model 1 (clinical risk factors + plaque characteristics), model 2 (clinical risk factors + presence of 2-PLD ATAs), and model 3 (clinical risk factors + plaque characteristics + presence of 2-PLD ATAs).

**Table 4:** Multivariate logistic analyses for ischemic stroke appearance.

	Univariable analysis		Multivariable analysis	
	OR (95 CI)	*p*-value	OR (95 CI)	*p-*value
Model 1				
Age (year)	0.97 (0.944–0.995)	0.022*		
Men	0.859 (0.424–1.739)	0.671		
Hypertension	2.977 (1.49–6.093)	0.002*	3.208 (1.214–8.995)	0.021*
Plaque burden	1.09 (1.058–1.128)	<0.001*		
Degree of stenosis	1.047 (1.026–1.071)	<0.001*		
Positive remodeling	5.843 (2.911–12.15)	<0.001*	3.966 (1.323–12.57)	0.016*
Intraplaque hemorrhage	6.279 (2.771–15.76)	0.002*	4.580 (1.534–15.45)	0.009*
Enhancement ratio	1.035 (1.022–1.05)	<0.001*	1.022 (1.008–1.039)	0.004*
Model 2				
Age (year)	0.97 (0.944–0.995)	0.022*		
Men	0.859 (0.424–1.739)	0.671		
Hypertension	2.977 (1.49–6.093)	0.002*	3.461 (1.376–9.215)	0.010*
1.5-s ATAs present	10.909 (4.62–29.14)	<0.001*	4.700 (1.673–14.44)	0.004*
2.5-s ATAs present	14.312 (6.54–33.72)	<0.001*	8.743 (3.617–22.69)	<0.001*
Model 3				
Age (year)	0.97 (0.944–0.995)	0.022*		
Men	0.859 (0.424–1.739)	0.671		
Hypertension	2.977 (1.49–6.093)	0.002*		
Plaque burden	1.09 (1.058–1.128)	<0.001*		
Degree of stenosis	1.047 (1.026–1.071)	<0.001*		
Positive remodeling	5.843 (2.911–12.15)	<0.001*	4.055 (1.243–14.52)	0.024*
Intraplaque hemorrhage	6.279 (2.771–15.76)	0.002*		
Enhancement ratio	1.035 (1.022–1.05)	<0.001*	1.018 (1.003–1.036)	0.024*
1.5-s ATAs present	10.909 (4.62–29.14)	<0.001*	4.604 (1.301–17.97)	0.021*
2.5-s ATAs present	14.312 (6.54–33.72)	<0.001*	4.950 (1.610–16.77)	0.007*

OR, odds ratio; CI, confidence interval; ATAs, arterial transit artifacts. Model 1, clinical risk factors + plaque characteristics; Model 2, clinical risk factors + 2-PLD ATAs present; and Model 3, clinical risk factors + plaque characteristics + 2-PLD ATAs present. * *p* < 0.05 indicates a significant difference.

**Figure 2:**
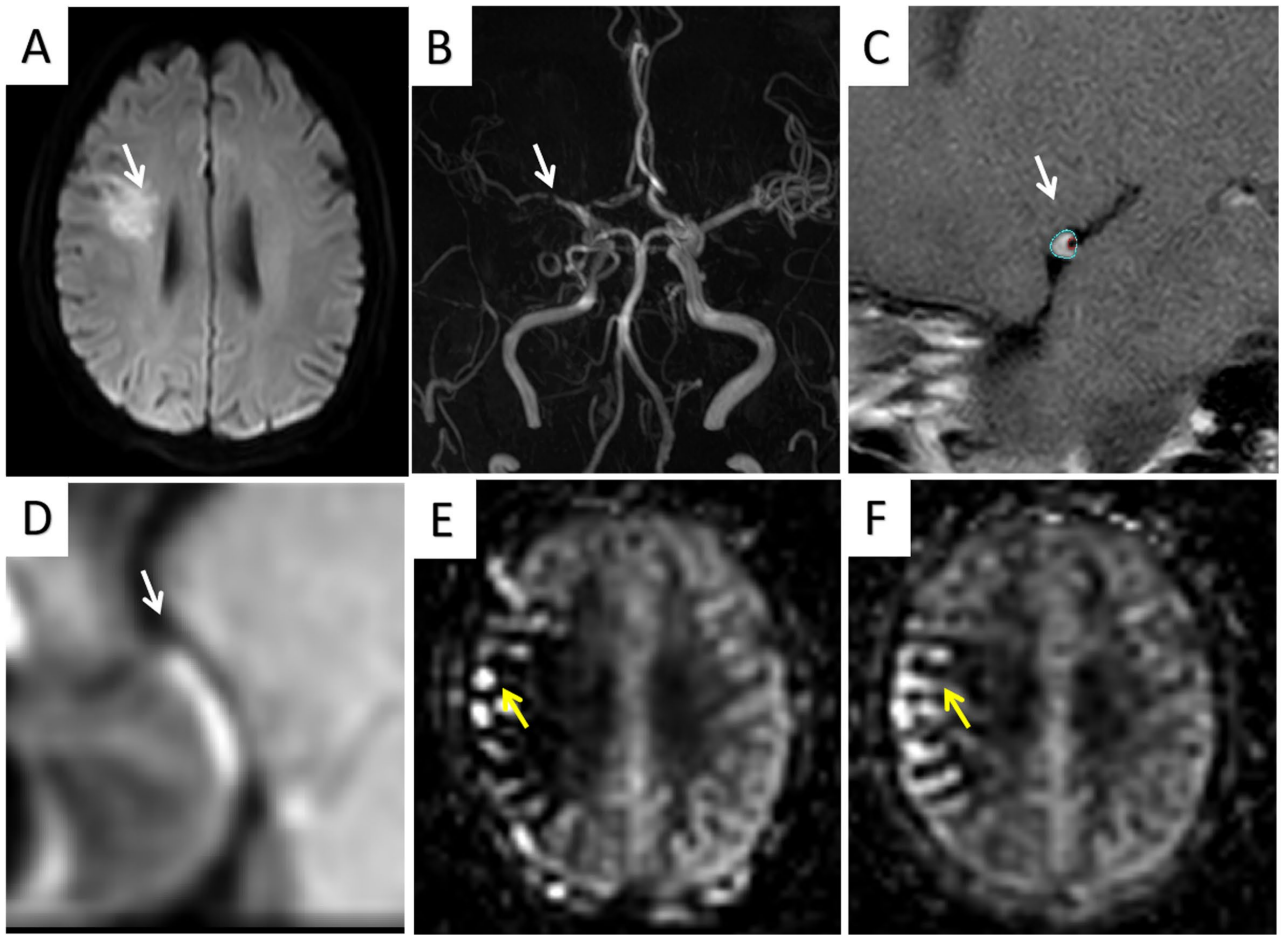
A stroke patient (age 73 years) showing a adjacent to the right lateral ventricle infarction **(A)** and right MCA stenosis **(B)**; HRMRI showed significant enhancement of the eccentric plaque **(C,D)**; ATA was present in the lesion area on 1.5-s ASL images **(E)**, and was still present in the lesion area, but with a reduced range, on 2.5-s ASL images **(F)**.

**Figure 3:**
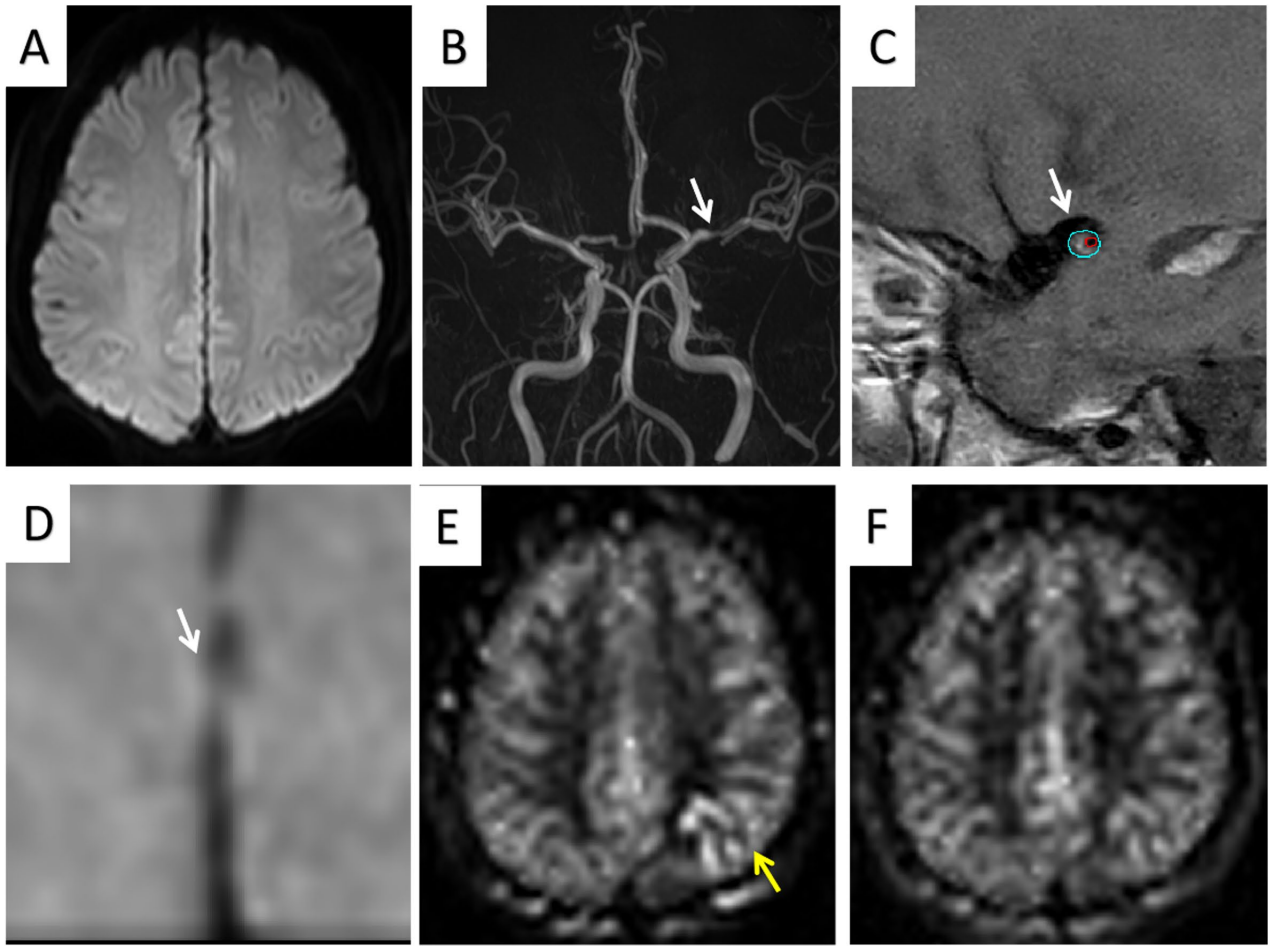
One TIA patient (age 67 years) showed no infarct area **(A)** and left MCA stenosis **(B)**; HRMRI showed moderate enhancement of the eccentric plaque **(C,D)**; ATA was present in the lesion area on 1.5-s ASL images **(E)** but was absent in the lesion area on 2.5-s ASL images **(F)**.

In the multivariate logistic regression analysis of model 1, hypertension (OR = 3.2088; 95% CI, 1.214–8.995), positive remodeling (OR = 3.966; 95% CI, 1.323–12.57), intraplaque hemorrhage (OR = 4.580; 95% CI, 1.534–15.45), and enhancement ratio (OR = 1.022; 95% CI, 1.008–1.039) were independent predictors of stroke events. In the multivariate logistic regression analysis of model 2, hypertension (OR = 3.461; 95% CI, 1.376–9.215) and the presence of 1.5-s ATAs (OR = 4.700; 95% CI, 1.673–14.44) and 2.5-s ATAs (OR = 8.743; 95% CI, 3.617–22.69) were independent predictors of stroke events. In the multivariate logistic regression analysis of model 3, positive remodeling (OR = 4.055; 95% CI, 1.243–14.52), enhancement ratio (OR = 1.018; 95% CI, 1.003–1.036), and the presence of 1.5-s ATAs (OR = 4.604; 95% CI, 1.301–17.97) and 2.5-s ATAs (OR = 4.950; 95% CI, 1.610–16.77) were independent predictors of stroke events (Table 4). Figures 2, 3 show a typical example of two groups of patients.

**Figure 4:**
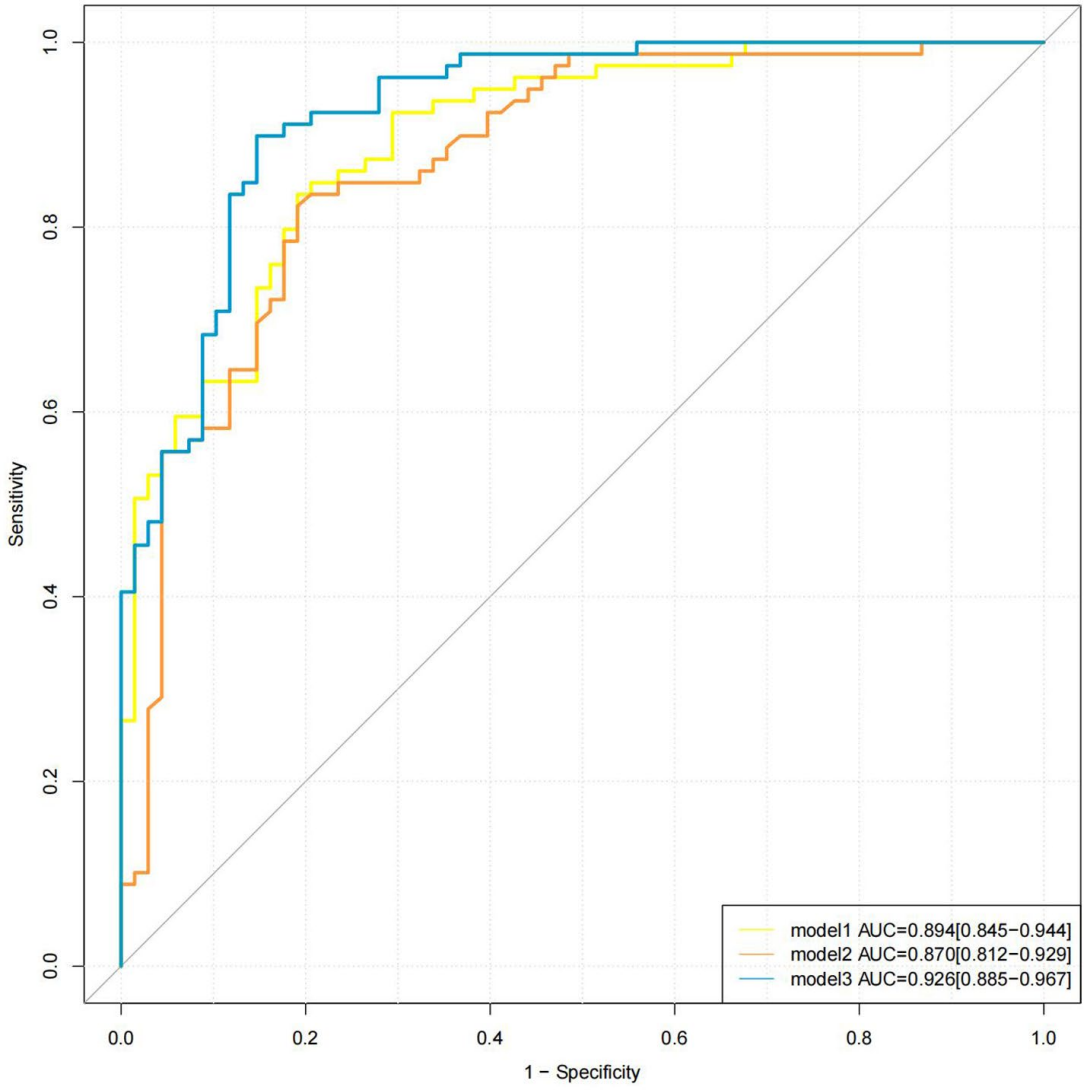
Different models for detection of stroke. The AUC values of models 1, 2, and 3 were 0.894, 0.870, and 0.926, respectively. AUC, area under the curve.

All three models showed excellent diagnostic performance in distinguishing between ischemic stroke and TIA. Model 3 (AUC, 0.926; 95% CI, 0.885–0.967) demonstrated superior diagnostic performance compared to Model 1 (AUC, 0.894; 95% CI, 0.845–0.944, Z = −1.982, *p* = 0.047) and Model 2 (AUC, 0.870; 95% CI, 0.812–0.929, Z = 2.552, *p* = 0.011), while no significant difference was observed between Model 1 and Model 2 (95% CI, −0.039 to 0.087, Z = 0.742, *p* = 0.458). The sensitivities of Model 1, Model 2, and Model 3 were 83.54, 82.28, and 89.87%, respectively; the specificities were 80.88, 80.88, and 85.29%; the positive predictive values were 83.54, 83.33, and 87.65%; the negative predictive values were 80.88, 79.71, and 87.88%; and the accuracies were 82.31, 81.63, and 87.76%, respectively. The ROC curves of the three models are shown in Figure 4.

## Discussion

The existing studies on stroke mainly focus on the structure and composition of plaques, and hemodynamic injuries have been less investigated (7, 8, 11). This study combined plaque and hemodynamic characteristics to explore the relationship between TIA and stroke patients, yielding three important findings: First, plaque characteristics (positive remodeling, intraplaque hemorrhage, and enhancement ratio) and the presence of 2-PLD ATAs were independently related to the incidence of stroke. Second, in comparison with HRMRI, the combination of 2-PLD ATAs with clinical risk factors showed no significant difference in distinguishing between patients with TIA and stroke. Third, the multimodal model that integrated positive remodeling, enhancement ratio, and the presence of 2-PLD ATAs demonstrated strong diagnostic performance in predicting stroke occurrence.

ATAs are caused by residual labeled blood in the supplying arteries that have not yet been distributed to microvessels and tissues. In this study, the stroke group exhibited a significantly higher incidence of 1.5-s and 2.5-s ATAs, indicating more severe hemodynamic impairment. A previous study using ASL found that ATAs were the only factor associated with recent ischemic symptoms in participants with carotid stenosis, while other plaque features were unrelated to symptom status (10). Thus, compared to plaques, ATAs are more indicative of symptomatic ischemic patients. Similarly, Liu et al. reported a significantly higher incidence of brain tissue volume with Tmax >6.0 s on DSC-PWI in the stroke group (11), suggesting that hypoperfusion can reduce blood flow in cortical or boundary areas, leading to arterio-arterial embolic infarctions. These findings align with the results of this study.

In Model 3, the presence of 1.5-s ATAs and 2.5-s ATAs was significantly associated with stroke. While ATAs can be corrected in multi-PLD acquisition, PLD changes help distinguish early hypersignal from true hyperperfusion in large vessels. However, this process is complex and challenging (15). ASL is particularly sensitive to hemodynamic changes, so delays in blood flow from the neck to the imaging area can lead to the appearance of ATAs. However, the presence of ATA has been associated with collateral circulation compensation (23). Our findings indicate that the presence of 1.5-s ATAs and 2.5-s ATAs are independently associated with stroke. This suggests that stroke patients experience longer blood flow delays to brain tissue, indicating more severe cerebral hemodynamic injury and a higher risk of infarction. ATAs not only provide information about collateral circulation but also reflect the severity of hemodynamic impairment. Consequently, prolonged PLD highlights the differences in blood flow abnormalities between TIA and stroke patients. ASL with multiple PLDs thus holds significant clinical value.

Another advantage of ASL over DSC-PWI is its use of endogenous water-molecule labeling instead of exogenous contrast agents (10). ATAs can be easily assessed through visual inspection of ASL-PWI scans, demonstrating good agreement and reproducibility in this study. Furthermore, combining clinical risk factors with ASL findings showed strong diagnostic efficacy in distinguishing stroke patients from those with TIA. Therefore, ATA assessments could become a widely used imaging marker in clinical practice.

HRMRI has become a valuable tool for directly assessing atherosclerotic plaques (24, 25). Numerous studies have utilized HRMRI to identify imaging biomarkers of symptomatic MCA plaques (26), providing unique radiological insights for diagnosing and treating ischemic stroke. Our study revealed significant differences in plaque morphology and composition between patients with ischemic stroke and those with TIA. These findings could advance research on the mechanisms of ischemic stroke and the potential links between TIA and stroke.

Our results indicated that luminal-positive remodeling, intraplaque hemorrhage, and plaque enhancement ratio were independently associated with stroke. Consistent with previous studies, these plaque characteristics are strong imaging biomarkers of patients with ischemic stroke (26). Furthermore, our findings revealed distinct pathological differences between stroke and TIA patients. Positive remodeling, a marker of plaque vulnerability, is associated with a higher likelihood of microemboli formation at the MLN site (27). Intraplaque hemorrhage, another independent risk factor for ischemic stroke, plays a significant role in the progression and recurrence of ischemic events. Plaque hyperintensity has been identified as a sign of intraplaque hemorrhage, often resulting from the rupture of thin-walled microvessels or vasotrophic vessels (28). Additionally, plaque enhancement may result from inflammation, neovascularization, or gadolinium leakage due to endothelial dysfunction (29). Lu et al. reported a significant correlation between the degree of MCA plaque enhancement and the presence of downstream acute infarction (30). Our findings indicated a higher incidence of positive remodeling, intraplaque hemorrhage, and an increased plaque enhancement ratio in the stroke group. This suggests that these patients may have more unstable plaques, a greater risk of plaque rupture, a higher tendency for microthrombosis formation in the lumen, and an elevated stroke risk. The plaque characteristics described above may have contributed to the abnormal distal blood flow that enhanced ATAs in stroke patients.

The strength of our study is the combination of two advanced MRI techniques (HRMRI and ASL) to simultaneously assess the differences in hemodynamic features and arterial plaque characteristics in stroke and TIA patients. Our results suggest that ASL may allow simpler detection of impaired cerebral hemodynamics and a better understanding of the differences in arterial plaque and hemodynamics underlying transient ischemic attack and stroke.

Nevertheless, this study has some limitations. First, selection bias may exist due to the cross-sectional study design. Second, the relatively small sample size necessitates larger studies to validate our findings. Third, certain important plaque features, such as plaque volume, fibrous cap, and lipid necrotic core, were not evaluated due to the small size of intracranial plaques and the challenges of accurate measurement. Finally, the lack of follow-up data limits our understanding of the relationship between these findings and patient outcomes. Future longitudinal studies are needed to confirm the stability and applicability of our model.

## Conclusion

A model incorporating plaque characteristics and ATAs demonstrated strong diagnostic performance in distinguishing TIA from stroke in patients with intracranial atherosclerotic stenosis. Compared to HRMRI, ASL offers a simpler imaging evaluation method, and the assessment of ATAs has the potential to become a widely adopted imaging marker in clinical practice.

## Data Availability

The original contributions presented in the study are included in the article/Supplementary material, further inquiries can be directed to the corresponding authors.

## Footnotes

^1^ tsimaging.net

## Supplementary material

The Supplementary material for this article can befound online at: https://www.frontiersin.org/articles/10.3389/fneur.2025.1514679/full#supplementary-material
