# Supplementary material for: Distinguishing stroke from transient ischemic attack using plaque characteristics and arterial transit artifacts
Source: Front Neurol. 2025 Mar 21;16:1514679. doi: 10.3389/fneur.2025.1514679 (PMC11968375; doi:10.3389/fneur.2025.1514679)
Supplement: Supplementary file 1 [file Table_1.docx]

Supplemental Table 1. Inter-reader agreement in measurement of plaque characteristics and evaluation of ATAs

| **Characteristics** | **ICC** | **P** |
| --- | --- | --- |
| Plaque burden | 0.816 (0.730-0.876) | < 0.001* |
| Degree of stenosis | 0.966 (0.947-0.978) | < 0.001* |
| Remodeling index | 0.827 (0.745-0.884) | < 0.001* |
| Eccentricity index | 0.881 (0.822-0.921) | < 0.001* |
| Enhancement ratio | 0.949 (0.923-0.967) | < 0.001* |
| Intraplaque hemorrhage | 0.796 (0.704-0.863) | < 0.001* |
| Enhanced grade | 0.935 (0.902-0.957) | < 0.001* |
| 1.5-s ATAs present | 0.845 (0.771-0.896) | 0.001* |
| 2.5-s ATAs present | 0.902 (0.852-0.936) | < 0.001* |
